# The hydrogen threshold of obligately methyl-reducing methanogens

**DOI:** 10.1093/femsle/fnaa137

**Published:** 2020-08-21

**Authors:** Christopher Feldewert, Kristina Lang, Andreas Brune

**Affiliations:** Research Group Insect Gut Microbiology and Symbiosis, Max Planck Institute for Terrestrial Microbiology, Marburg, Germany; Research Group Insect Gut Microbiology and Symbiosis, Max Planck Institute for Terrestrial Microbiology, Marburg, Germany; Research Group Insect Gut Microbiology and Symbiosis, Max Planck Institute for Terrestrial Microbiology, Marburg, Germany

**Keywords:** hydrogen, threshold, methanol, *Methanosphaera*, *Methanimicrococcus*, *Methanomassiliicoccus*

## Abstract

Methanogenesis is the final step in the anaerobic degradation of organic matter. The most important substrates of methanogens are hydrogen plus carbon dioxide and acetate, but also the use of methanol, methylated amines, and aromatic methoxy groups appears to be more widespread than originally thought. Except for most members of the family *Methanosarcinaceae*, all methylotrophic methanogens require external hydrogen as reductant and therefore compete with hydrogenotrophic methanogens for this common substrate. Since methanogenesis from carbon dioxide consumes four molecules of hydrogen per molecule of methane, whereas methanogenesis from methanol requires only one, methyl-reducing methanogens should have an energetic advantage over hydrogenotrophic methanogens at low hydrogen partial pressures. However, experimental data on their hydrogen threshold is scarce and suffers from relatively high detection limits. Here, we show that the methyl-reducing methanogens *Methanosphaera stadtmanae* (*Methanobacteriales*), *Methanimicrococcus blatticola* (*Methanosarcinales*), and *Methanomassiliicoccus luminyensis* (*Methanomassiliicoccales*) consume hydrogen to partial pressures < 0.1 Pa, which is almost one order of magnitude lower than the thresholds for *M. stadtmanae* and *M. blatticola* reported in the only previous study on this topic. We conclude that methylotrophic methanogens should outcompete hydrogenotrophic methanogens for hydrogen and that their activity is limited by the availability of methyl groups.

## Teaser

Based on their extremely low hydrogen threshold, methylotrophic methanogens (MM) should always outcompete hydrogenotrophic methanogens (HM) for hydrogen, provided that methyl groups are available in sufficient amounts.

## INTRODUCTION

Increasing atmospheric levels of methane have intensified the interest in understanding the sources of this second-most important greenhouse gas (Mikaloff, Fletcher and Schaefer 2019). About half of the global methane emission is biogenic, stemming mostly from natural wetlands and sediments, and to an increasing extent from agriculture and ruminant livestock (Kirschke *et al*. 2013). Methanogenesis is catalyzed by methanogenic archaea, which utilize either hydrogen plus carbon dioxide or acetate, the major products of microbial fermentations, as substrates (Liu and Whitman 2008; Thauer *et al*. 2008). However, also methylated compounds are emerging as an important group of methanogenic substrates (Evans *et al*. 2019; Söllinger and Urich 2019). While the contribution of hydrogenotrophic and aceticlastic methanogenesis to methane production has been intensively studied, the importance of methylotrophic methanogenesis remains unclear (Conrad 2020).

Hydrogenotrophic methanogens reduce carbon dioxide to methane via the archaeal variant of the Wood–Ljungdahl pathway. The key intermediate is methyl-coenzyme M, whose formation by a membrane-bound methyl-H_4_MPT:coenzyme M methyltransferase is highly exergonic and allows the conservation of energy in the form of an electrochemical sodium gradient (Thauer *et al*. 2008). By contrast, methylotrophic methanogens transfer the methyl groups of methanol or other methylated substrates directly to coenzyme M (Thauer *et al*. 2008; Yan and Ferry 2018). This bypasses the methyltransferase reaction and therefore requires other modes of energy conservation (Sprenger, Hackstein and Keltjens 2005; Fricke *et al*. 2006; Lang *et al*. 2015).

Also methylotrophic methanogens generally require molecular hydrogen for methanogenesis. Only the methyl-fermenting members of the family *Methanosarcinaceae*, which possess a complete Wood–Ljungdahl pathway and a membrane-bound electron transport chain, can generate reducing equivalents for methanogenesis by oxidizing methyl groups to carbon dioxide (Thauer *et al*. 2008). Obligately methyl-reducing methanogens comprise phylogenetically and biochemically heterogeneous lineages, including the isolates *Methanosphaera stadtmanae* (*Methanobacteriales*; Miller and Wolin 1985), *Methanimicrococcus blatticola* (*Methanosarcinales*; Sprenger *et al*. 2000), and *Methanomassiliicoccus luminyensis* (*Methanomassiliicoccales*; Didri *et al*. 2012), the recently described *Methanonatronarchaeum thermophilum* (*Methanonatronarchaeales*; Sorokin *et al*. 2018) and several *Candidatus* strains from enrichment cultures (Borrel *et al*. 2012; Paul *et al*. 2012; Borrel *et al*. 2013; Iino *et al*. 2013; Sorokin *et al*. 2018). Also a few hydrogenotrophic *Methanobacterium* species can facultatively reduce methanol (Krivushin *et al*. 2010; Borrel *et al*. 2012). Evidence for an even wider range of putatively hydrogen-dependent, methylotrophic methanogens is provided by a growing number of metagenome-assembled genomes of uncultured archaea (Evans *et al*. 2019; Söllinger and Urich 2019).

Under standard conditions, hydrogenotrophic methanogenesis (Equation 1) is thermodynamically more favorable than the hydrogen-dependent reduction of methanol to methane (Equation 2) (Thauer 1998).

(1) $$\begin{eqnarray*}
{\rm 4\,H}_{\rm 2} + {\rm CO}_{\rm 2} \to {\rm CH}_{\rm 4} + {\rm 2H}_{\rm 2}{\rm O}\;\;\;({\rm \Delta G}^{\circ\,\prime} = -131\, {\rm kJ\, mol}^{\rm-1}\,{\rm methane})
\end{eqnarray*}$$

(2) $$\begin{eqnarray*}
{\rm H}_{\rm 2} + {\rm CH}_{\rm 3}{\rm OH} \to {\rm CH}_{\rm 4} + {\rm H}_{\rm 2}{\rm O}\;\;\;({\rm \Delta G^{\circ\,\prime} = -112.5\, {\rm kJ\,mol}^{\rm-1}\,{\rm{methane}}})\nonumber\\
\end{eqnarray*}$$

However, the different stoichiometries of the reactions dictate that the free energy of hydrogenotrophic methanogenesis decreases more strongly with decreasing hydrogen partial pressure, and methyl-reducing methanogens should eventually outcompete hydrogenotrophic methanogens for their common substrate.

Hydrogen thresholds of hydrogenotrophic methanogens have been investigated in a number of studies. In most cases, the threshold value ranges between 2.8 and 10 Pa (Table 1). An exception is members of the genus *Methanosarcina*, which exhibit considerably higher threshold values for hydrogen when grown hydrogenotrophically (Table 1) and even accumulate hydrogen at steady-state levels of 8–20 Pa during fermentation of acetate, methanol or methylamines by hydrogen cycling (e.g. Lovley and Ferry 1985; Kulkarni, Mand and Metcalf 2018). Data on obligately methyl-reducing methanogens, however, are scarce. The only hydrogen threshold values reported in the literature (1.0 Pa for *Methanosphaera stadtmanae* and < 0.7 Pa for *Methanimicrococcus blatticola*) are around the detection limit given in that study (Sprenger, Hackstein and Keltjens 2007). Therefore, we re-investigated the hydrogen thresholds of these species and of the hitherto unstudied *Methanomassiliicoccus luminyensis*, the only isolate of the exclusively methyl-reducing *Methanomassiliicoccales*, using an analytical setup that was more sensitive by almost one order of magnitude (detection limit 0.1 Pa). A hydrogenotrophic *Methanobrevibacter* species was included for benchmarking purposes.

**Table 1.** Hydrogen thresholds of hydrogenotrophic and methyl-reducing methanogens from different orders determined in this and previous studies.

Order	Species	Hydrogen threshold (Pa)^a^	Reference
***Hydrogenotrophic***			
*Methanobacteriales*	*Methanobacterium bryantii* strain M.o.H.	6.9 ± 1.5 0.056 (0.028–0.14)^b^	Lovley (1985) Karadagli and Rittmann (2007)
	*Methanobacterium formicicum* strain JF-1	6.5 ± 0.6	Lovley (1985)
	*Methanobacterium formicicum* strain DSM 1535	2.8	Cord-Ruwisch *et al*. (1988)
	*Methanobacterium formicicum* ^c^	4.5	Kral *et al*. (1996)
	*Methanobacterium bryantii* strain DSM 10 113	2.5 ± 4.4^d^	Neubeck *et al*. (2016)
	*Methanobrevibacter arboriphilus* strain DSM 744	9.0	Cord-Ruwisch *et al*. (1988)
	*Methanobrevibacter smithii* strain DSM 816	10.0	Cord-Ruwisch *et al*. (1988)
	*Methanobrevibacter* strain AMG-1	5.7 ± 0.7	This study
*Methanomicrobiales*	*Methanospirillum hungatei* strain JF-1	9.5 ± 1.3	Lovley (1985)
	*Methanospirillum hungatei* strain DSM 864	3.0	Cord-Ruwisch *et al*. (1988)
	*Methanoculleus bourgensis* strain MAB1	0.15 ± 0.13^d^^,^^e^	Neubeck *et al*. (2016)
*Methanococcales*	*Methanococcus vannielii* strain DSM 1224	7.5	Cord-Ruwisch *et al*. (1988)
*Methanosarcinales*	*Methanosarcina barkeri* ^c^	15.7 ± 2.7	Kral *et al*. (1996)
	*Methanosarcina barkeri* strain DSM 800	18.6 ± 10.0	Neubeck *et al*. (2016)
***Methyl-reducing***			
*Methanobacteriales*	*Methanosphaera stadtmanae* strain DSM 3091	1.0 ± 0.3^e^	Sprenger, Hackstein and Keltjens (2007)
		< 0.1^f^	This study
*Methanosarcinales*	*Methanimicrococcus blatticola* strain DSM 13 328	< 0.7^f^	Sprenger, Hackstein and Keltjens (2007)
		< 0.1^f^	This study
*Methanomassiliicoccales*	*Methanomassiliicoccus luminyensis* strain DSM 25 720	< 0.1^f^	This study

a All values were converted to partial pressures and are given with the standard deviations reported in the respective studies.

b Partial pressures recalculated from dissolved H_2_ with the conversion factor used by Karadagli and Rittmann (2007).

c No strain specified; cultures obtained from David R. Boone.

d Values for standard medium.

e Around the detection limit reported in the respective study.

f Below the detection limit reported in the respective study.

## MATERIAL AND METHODS

*Methanosphaera stadtmanae* (DSM 3091), *Methanimicrococcus blatticola* (DSM 13 328), and *Methanomassiliicoccus luminyensis* (DSM 25 720) were obtained from Deutsche Sammlung von Mikroorganismen und Zellkulturen (DSMZ). The obligately hydrogenotrophic *Methanobrevibacter* strain AMG-1, a member of the *Methanobrevibacter arboriphilus* clade (Genbank accession number MT249795; 94.5% 16S rRNA gene sequence similarity to the type strain), was isolated from the gut of the millipede *Anadenobolus monilicornis* (C. Netz, K. Lang and A. Brune, unpublished results). All strains were grown in AM5 medium (Tegtmeier *et al*. 2016) supplemented with (final concentration) 2-mercaptoethane sulfonate (0.001%), casamino acids (0.2%; Roth), yeast extract (0.2%; Roth), cysteine-HCl (2 mM), acetate (1 mM), dithiothreitol (1 mM), formate (0.5 mM) and methanol (50 mM). Serum bottles (120 ml) filled with 30 ml medium under a headspace of N_2_–CO_2_ (80/20) were inoculated with 3 ml preculture (three replicates). After addition of hydrogen (150 Pa) to the headspace, the cultures were incubated statically at their optimum growth temperatures (37°C for *M. stadtmanae* and *M. luminyensis*, and 30°C for *M. blatticola* and *Methanobrevibacter* strain AMG-1). The amount of methanol in the medium (1.5 mmol) by far exceeded the amount of hydrogen added to the cultures (5.6 µmol per addition).

Hydrogen partial pressures were monitored once per week. Aliquots (0.2 ml) of the headspace were injected into a gas chromatograph (GC 8A, Shimadzu, Kyoto, Japan) equipped with a Molsieve column (60/80 mesh, 6 ft length, 2.1 mm inner diameter, Restek, Bad Homburg, Germany; column temperature 80°C) and a reducing gas detector for hydrogen partial pressures < 10 Pa (RGD2, Trace Analytical, Techmation, Düsseldorf, Germany). Hydrogen partial pressures > 10 Pa were measured with a gas chromatograph equipped with a thermal conductivity detector. Standard curves were generated by injecting aliquots (0.1, 0.2 and 0.4 ml) of hydrogen standards (2 ppm and 1000 ppm, in N_2_; Messer, Bad Soden, Germany). All injections were carried out with a gas-tight 0.5-ml precision syringe (Grace Davison Discovery Science, Deerfield, IL, USA). The detection limit of the assay was defined as the amount of hydrogen that caused a peak that had twice the height of the baseline noise at the retention time of hydrogen when a non-reducing gas (nitrogen) was injected. To monitor growth of the precultures, methanogenesis was routinely measured using a separate gas chromatograph equipped with a flame ionization detector (Lang *et al*. 2015).

## RESULTS AND DISCUSSION

**Figure 1.**
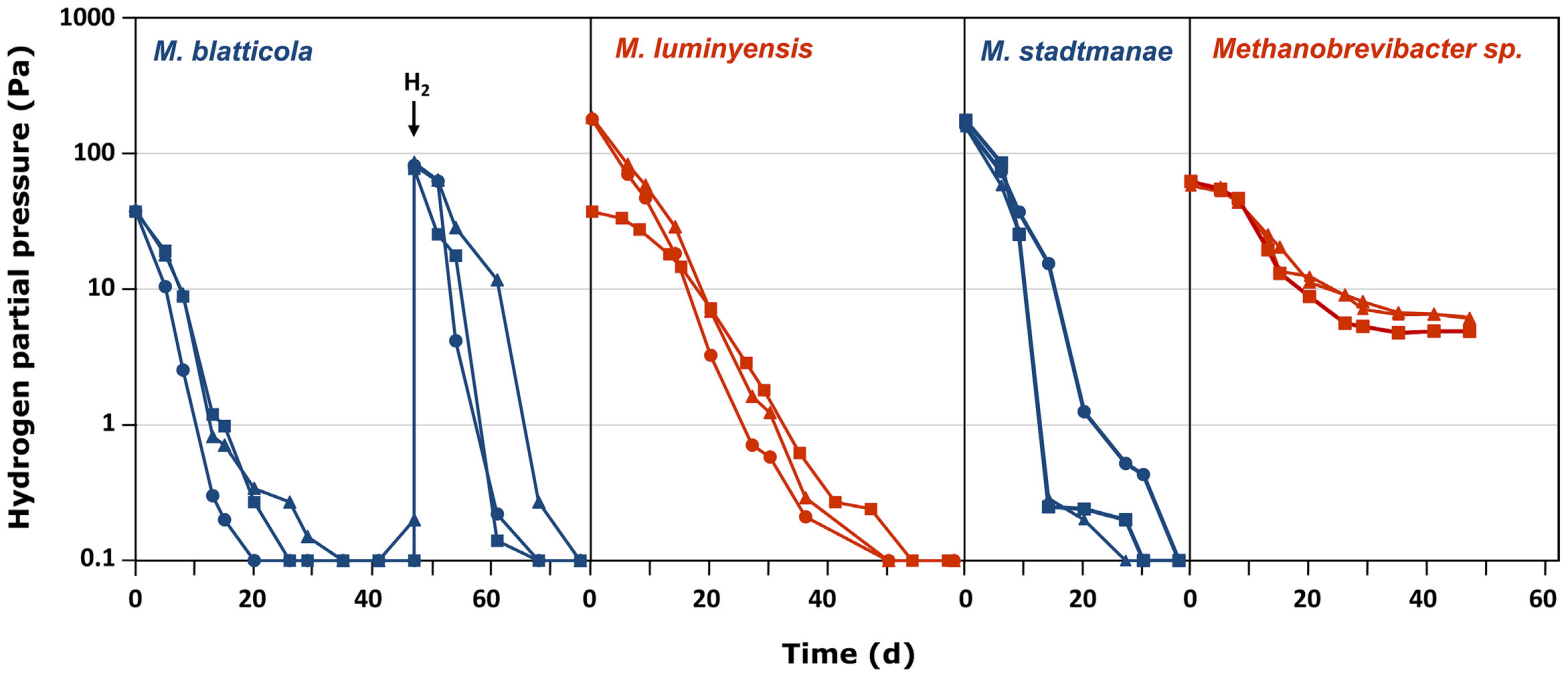
Time course of hydrogen consumption by *Methanimicrococcus blatticola*, *Methanomassiliicoccus luminyensis*, *Methanosphaera stadtmanae* and *Methanobrevibacter* strain AMG1 (three replicate cultures). The baseline represents the detection limit of our assay system (0.1 Pa). The arrow in the graph of *M. blatticola* exemplifies the addition of fresh hydrogen to the culture (not shown for all species).

All cultures consumed the hydrogen added to the headspace. The hydrogenotrophic *Methanobrevibacter* strain AMG-1 consumed hydrogen until a threshold value of 5.7 ± 0.7 Pa was reached. The three obligately methyl-reducing methanogens, *Methanosphaera stadtmanae*, *Methanimicrococcus blatticola* and *Methanomassiliicoccus luminyensis*, however, always consumed hydrogen to partial pressures that were below the detection limit of our assay system (0.1 Pa; Fig. 1). When hydrogen was added again, all strains immediately resumed hydrogen consumption, which indicated that the cultures remained metabolically active.

The hydrogen threshold of *Methanobrevibacter* strain AMG-1 is about 40% lower than the values reported for *Methanobrevibacter arboriphilus* and *Methanobrevibacter smithii* (9–10 Pa, Cord-Ruwisch, Seitz and Conrad 1988), but within the range reported for other hydrogenotrophic methanogens (2.5–6.9 Pa; Table 1). The hydrogen thresholds of the three methyl-reducing species, although not determined exactly, are definitely below our detection limit (i.e. < 0.1 Pa). This value is up to one order of magnitude lower than the hydrogen thresholds reported for *M. stadtmanae* and *M. blatticola* in the only previous study on this topic. The discrepancy is explained by the higher detection limit of the assay system used by Sprenger, Hackstein and Keltjens (2007), which was at or close to the reported threshold levels.

These hydrogen thresholds are consistent with thermodynamics. Assuming standard conditions for all other reactants, the thermodynamic equilibrium of hydrogenotrophic methanogenesis (Equation 1) will be reached at a hydrogen partial pressure of 0.18 Pa (Thauer *et al*. 2008). In methanogens without cytochromes, which have low growth yields and may gain as little as 0.3 ATP per methane (e.g. *Methanobrevibacter arboriphilus*), energy metabolism and ATP synthesis would be in equilibrium at a hydrogen partial pressure of 1 Pa. Methanogens with cytochromes, however, are far more efficient in energy conservation (1.5 ATP per methane for *Methanosarcina barkeri*), and their theoretical hydrogen threshold should be more than one order of magnitude higher (Thauer *et al*. 2008). These considerations are in agreement with the experimental thresholds (Table 1), except for the values reported for *Methanobacterium bryantii* (Karadagli and Rittmann 2007) and *Methanoculleus bourgensis* (Neubeck *et al*. 2016), which are close to or even lower than those expected at the thermodynamic equilibrium.

Using the same theoretical framework, we found that also the much lower hydrogen threshold of methylotrophic methanogens matches theoretical expectations. Assuming standard conditions for all reactants but hydrogen, and a phosphorylation potential of 50 kJ per mol ATP (Thauer *et al*. 2008), methyl-reducing methanogenesis (Equation 2) would be in thermodynamic equilibrium at a hydrogen partial pressure of 1.8 × 10^–15^ Pa. At a methanol concentration of 50 mM (experimental conditions), which decreases the free energy change of the reaction to –105.1 kJ mol^–1^, the theoretical hydrogen threshold of methylotrophic methanogenesis ranges between 3.5  **×  **10^–11^ Pa (at 0.3 ATP per methane) and 0.5 Pa (at 1.5 ATP per methane). Even at a methanol concentration of 10 µM, which decreases the free energy change of the reaction to –84.5 kJ mol^–1^, a methylotrophic methanogen should still be able to synthesize 1 ATP per methane at a hydrogen partial pressure of 0.1 Pa. It is important to note that due to the reaction stoichiometry, the hydrogen threshold value of methyl-reducing methanogens will be affected more strongly by their actual ATP gain per mol of methane than that of hydrogenotrophic methanogens.

Although all methyl-reducing methanogens studied to date employ the same biochemistry of methane formation, their modes of heterodisulfide reduction and energy conservation differ fundamentally between members of different orders. Like other methanogens without cytochromes, *Methanosphaera stadtmanae* uses a soluble hydrogenase/heterodisulfide reductase complex (MvhADG/HdrABC) to regenerate the coenzymes and to produce reduced ferredoxin by electron bifurcation; the free energy of the reduced ferredoxin is harvested with an energy-converting hydrogenase (Ehb complex) in the form of an electrochemical sodium gradient (Thauer *et al*. 2008). *Methanimicrococcus blatticola*, a methanogen with cytochromes, uses an electron transport chain consisting of a hydrogenase (VhoACG) coupled to a membrane-bound heterodisulfide reductase (HdrDE) via a phenazine carrier (Sprenger, Hackstein and Keltjens 2005). A similar strategy is probably used by the extremely halophilic *Methanonatronarchaeum thermophilum* (Sorokin *et al*. 2017), the only obligately methyl-reducing methanogen available in pure culture that was not included in the present study. *Methanomassiliicoccus luminyensis*, which lacks both energy-converting hydrogenases and cytochromes, employs a new mode of energy conservation that combines both strategies. One heterodisulfide formed during methanogenesis is reduced by the MvhADG/HdrABC complex, producing reduced ferredoxin. A second heterodisulfide is reduced by a membrane-bound Fpo-like 11-subunit complex that is presumably associated with HdrD and generates an electrochemical proton potential (Lang *et al*. 2015; Kröninger et al. 2016, 2019).

The exact ATP gains achieved by these different modes of energy conservation are not known, but the growth yields on hydrogen and methanol (dry weight; Equation 2) reported for *Methanosphaera stadtmanae* (∼4 g per mol methane; Miller and Wolin 1985) and *Methanimicrococcus blatticola* (3.5–6.0 g per mol methane) are in the same range as that of *Methanosarcina barkeri* (4.6 g per mol methane in methyl-reducing and 6.5 g per mol methane in methyl-fermenting cultures; Müller, Blaut and Gottschalk 1986). Growth yields for *Methanomassiliicoccus luminyensis* are much lower (2.4 g per mol methane; Kröninger, Gottschling and Deppenmeier 2017). This agrees with the metabolic model that was previously proposed for *Methanomassiliicoccales*, which predicts the translocation of 3–4 protons per two molecules of methane (Lang *et al*. 2015, Kröninger *et al*. 2016). This would yield only about 0.5 ATP per methane, which is in the same range as in hydrogenotrophic methanogens and may allow an even lower hydrogen threshold than in other methyl-reducing species.

While methyl-reducing methanogens compete with hydrogenotrophic methanogens for hydrogen, they compete with methyl-fermenting methanogens for methanol and other methylated substrates. Based on the amount of methane produced, methanol fermentation (Equation 3) yields almost the same amount of free energy as methanol reduction with hydrogen (Equation 2) under standard conditions, but due to reaction stoichiometry, it is more sensitive to changes in methanol concentration.

(3) $$\begin{eqnarray*}
&& {\rm 4 CH}_{\rm 3}{\rm OH} \to {\rm 3 CH}_{\rm 4} + {\rm CO}_{\rm 2} + {\rm 2}{\rm H}_{\rm 2}{\rm O}\nonumber\\
&&\quad\;\;\;\;({\rm \Delta G}^{\circ\,\prime} = -106.5\, {\rm kJ\,mol}^{\rm -1}\,{\rm methane})
\end{eqnarray*}$$

At the low methanol concentrations encountered in cockroach guts (10 µM; Sprenger, Hackstein and Keltjens 2007), methanol reduction (ΔG′ = –84 kJ/mol) is considerably more exergonic than methanol fermentation (ΔG′ = –67 kJ/mol; all other reactants at standard conditions) and remains energetically more favorable at hydrogen partial pressures down to 100 Pa. This matches the substrate affinities for methanol of *Methanimicrococcus blatticola* and *Methanosphaera stadmanae* (*K*_S_ = 5–20 µM), which are much higher than those of *Methanosarcina barkeri* (*K*_S_ = 180–250 µM), and explains why *Methanomicrococcus blatticola* dominates the community of methylotrophic methanogens in cockroach guts (Sprenger, Hackstein and Keltjens 2007).

It is intriguing that most methyl-reducing methanogens studied to date (see above) have been enriched or isolated from the intestinal tracts of animals. They are conspicuously abundant in the intestinal tracts of millipedes and insects, the rumen of cows and sheep, and the colon of mammals (e.g. Henderson *et al*. 2013; Conway de Macario and Macario 2018; Brune 2019)—environments that are characterized by relatively high hydrogen partial pressures. This suggests that at least in these environments, their ecological success may not be based on their competitiveness for hydrogen but on their ability to utilize methyl groups at concentrations that are not accessible to methyl-fermenting methanogens. The obligately methyl-reducing *Methanimicrococcus blatticola* colonizes the hindgut of cockroaches feeding on pectin (Sprenger, Hackstein and Keltjens 2007), and selective feeding of termites with xylan, another plant cell wall component rich in methyl groups, increases the relative abundance of uncultured, putatively methyl-reducing *Methanomassilliicoccales* (Miyata *et al*. 2007). Also, lignin-derived methoxylated aromatic compounds are demethylated by the hindgut microbiota of termites (Brune, Miambi and Breznak 1995). The organisms responsible for this activity are not known, but the capacity for this reaction has been demonstrated in the methyl-disproportionating *Methermicoccus shengliensis* (*Methanosarcinales*), an isolate from coal beds (Mayumi *et al*. 2016). Another abundant substrate source for methylotrophic methanogens in intestinal environments is methylamines (Poulsen *et al*. 2013; Gaci *et al*. 2014).

Methanol and methylamines drive methanogenesis also in organic-rich marine and estuarine sediments, where methanogens are outcompeted for acetate and hydrogen by sulfate reducing bacteria (Oremland and Polcin 1982; Oremland, Marsh and Polcin 1982). Originally, the utilization of such ‘non-competitive’ substrates had been attributed to methyl-fermenting methylotrophs (e.g. *Methanosarcina* and *Methanococcoides* spp.; Lyimo *et al*. 2009), but the hydrogen thresholds of sulfate reducers (in the range of 1 Pa; Cord-Ruwisch et al. 1988; Ozuolmez et al. 2015) would permit methyl-reducing methanogens to utilize hydrogen even in the presence of sulfate if the concentration of methyl groups is sufficient. This agrees with the recent finding that metagenome-assembled genomes (MAGs) of uncultured, putatively methyl-reducing *Methanomassiliicoccales* and *Methanofastidiosa* represent the most active methanogens in coastal mangrove sediments (Zhang *et al*. 2020). Further evidence for the presence of putatively methyl-reducing methanogens in other environments (Evans *et al*. 2015; Nobu *et al*. 2016; Vanwonterghem *et al*. 2016; Sorokin *et al*. 2018; Berghuis *et al*. 2019; Liu *et al*. 2020) underscores that (hydrogen-dependent) methylotrophic methanogenesis is widespread and—due to the high affinity for hydrogen—most likely limited by the availability of methyl groups.
